# Clock Drawing Test Performance in Rural Vietnam: Reliability and Concurrent Validity Relative to the Mini‐Mental State Examination in a Community‐Based Sample

**DOI:** 10.1111/ggi.70876

**Published:** 2026-11-03

**Authors:** Aki Yazawa, Thuy Thi Phuong Pham, Chau Que Nguyen, Aoi Ito, Danh Cong Phan, Masahiko Hachiya, Thanh Tien Hoang, Hung Thai Do, Tetsuya Mizoue, Yosuke Inoue

**Affiliations:** ^1^ Department of Primary Care and Medical Education Institute of Medicine, University of Tsukuba Ibaraki Japan; ^2^ Department of Disease Prevention Pasteur Institute in Nha Trang Nha Trang Vietnam; ^3^ Department of Epidemiology and Prevention Center for Clinical Sciences, Japan Institute for Health Security Tokyo Japan; ^4^ Department of Environmental and Occupational Health Pasteur Institute in Nha Trang Nha Trang Vietnam; ^5^ Pasteur Institute in Nha Trang Nha Trang Vietnam; ^6^ Bureau of Global Health Cooperation, Japan Institute for Health Security Tokyo Japan

**Keywords:** Clock Drawing Test, cognitive screening, dementia, educational attainment, low‐ and middle‐income countries

## Abstract

**Aim:**

Some cognitive screening tools can be difficult to administer in population‐based epidemiological studies because they are time‐consuming and rely on familiarity with formal testing. The Clock Drawing Test (CDT) is a brief, pragmatic tool requiring minimal verbal instruction, but its performance in low‐ and middle‐income countries remains unclear. We examined the reliability and performance characteristics of the CDT and its concurrent validity relative to the Mini‐Mental State Examination (MMSE) performance in rural Vietnam.

**Methods:**

Data came from 1073 adults aged 55–65 years participating in the second wave (2023) of the Khánh Hòa Cardiovascular Study. The CDT was scored using the 6‐point Shulman method (lower scores indicate better performance). Inter‐rater reliability was assessed using intraclass correlation coefficients (ICC). Construct validity was examined via Spearman's correlations with MMSE scores. Known‐groups validity was tested across education levels. Concurrent validity relative to MMSE performance was evaluated by examining the discriminatory performance of the CDT using ROC analysis, sensitivity, specificity, predictive values, and agreement between CDT‐ and MMSE‐based classifications using Cohen's *κ*.

**Results:**

Inter‐rater reliability between two raters was good (single‐measure ICC = 0.79), and CDT scores correlated negatively with MMSE (*ρ* = −0.30, *p* < 0.001). CDT performance varied by education (*p* < 0.001). ROC analysis showed modest discriminatory accuracy (AUC = 0.68); a cutoff ≥ 3 yielded sensitivity 67% and specificity 62%, with limited agreement between CDT and MMSE classifications (*κ* = 0.13).

**Conclusions:**

In rural Vietnam, the CDT demonstrated acceptable reliability and modest concurrent validity relative to MMSE performance. Agreement between CDT‐ and MMSE‐based classifications was limited, suggesting that the two measures should not be considered interchangeable. Although a high negative predictive value was observed in this community‐based sample, its implications for cognitive screening remain uncertain. Further validation against clinical diagnostic outcomes and in higher‐risk populations is warranted.

## Graphical abstract

In 1073 older adults in rural Khanh Hoa, Vietnam, the Clock Drawing Test showed modest discrimination for low cognitive performance defined as MMSE ≤ 23 (AUC 0.68). Although the negative predictive value was high (94%), its utility for cognitive screening remains uncertain.

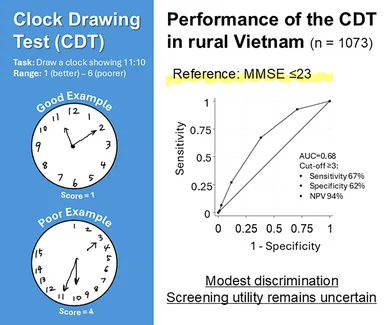

## 1 Introduction

Cognitive impairment and dementia are growing public health challenges worldwide, particularly in low‐ and middle‐income countries (LMICs), where populations are rapidly aging [1]. While early identification of cognitive decline is essential for planning care, initiating preventive interventions, and maintaining quality of life [2, 3], some commonly used screening instruments, such as the Mini‐Mental State Examination (MMSE) [4], are relatively lengthy and verbally demanding and may be difficult to administer in large‐scale epidemiological studies, particularly in populations with diverse educational backgrounds or limited familiarity with formal testing procedures [5].

The Clock Drawing Test (CDT), which requires individuals to draw a clock showing the time 11:10, has been widely used as a simple and rapid screening tool to assess visuospatial and executive functioning [6]. Beyond its brevity, the CDT offers several practical advantages for community‐based screening: It requires minimal equipment, is easy to administer and remember, and is generally perceived as less threatening or burdensome than longer verbal assessments. Because it places relatively limited demands on reading and writing, it may be applicable across diverse populations, including individuals with limited formal education or sensory impairments, making it potentially useful in field settings [7].

Despite these advantages, the extent to which the CDT translates into real‐world performance in population‐based settings has not been fully established. Previous studies, primarily conducted in high‐income countries, have reported moderate‐to‐strong correlations between CDT and MMSE scores and have demonstrated that CDT performance is strongly influenced by educational attainment [7, 8]. A systematic review found that a higher educational level was associated with better CDT performance in approximately 77% of analyses, and no or limited formal schooling negatively affected performance in nearly all studies [8]. However, evidence from LMICs, particularly from community‐based populations, remains limited. Existing studies from LMICs suggest that educational exposure, familiarity with formal testing, and cultural context may substantially influence CDT performance [8, 9]. Furthermore, few studies have evaluated the measurement properties of the CDT in rural Southeast Asian populations, where educational attainment often varies markedly across generations and between urban and rural areas. In Vietnam, older adults living in rural communities experienced substantial historical differences in access to formal education, providing an important setting in which to examine how the CDT performs under real‐world epidemiological survey conditions. To our knowledge, no previous study has comprehensively evaluated the measurement properties of the CDT in a large community‐based sample of older adults from rural Vietnam, a setting characterized by substantial heterogeneity in educational attainment and limited evidence on cognitive screening instruments.

Therefore, the present study aimed to examine the performance of the CDT relative to MMSE performance among community‐dwelling middle‐aged and older adults in rural Vietnam. We sought to characterize the CDT's reliability and multiple aspects of validity, including construct validity, known‐groups validity, and concurrent validity relative to MMSE performance, under conditions typical of large‐scale epidemiological surveys [10].

## 2 Methods

### 2.1 Study Participants

Data were obtained from the ongoing Khánh Hòa Cardiovascular Study (KHCS), a prospective cohort of 3000 middle‐aged adults designed to identify determinants of cardiovascular diseases in rural Vietnam [11, 12]. The baseline survey was conducted between 2019 and 2020 across eight communes in Khánh Hòa Province, Vietnam. Participants were recruited following an enumeration of residents aged 40–59 years who had lived in the communes for at least 6 months at the time of enrollment. Sociodemographic information was obtained at the baseline survey.

The present analysis used data from the second wave of the cohort conducted in 2023 and 2024, which included cognitive function assessments. Of the 3000 participants at baseline, 2562 participated in the second survey (follow‐up rate: 85.4%). A total of 438 participants were lost to follow‐up, including 44 deaths and 394 individuals who did not participate in the second survey. Among the follow‐up participants, 1198 were aged 55 years or older and were eligible for MMSE assessment according to the study protocol. Individuals who were unable to complete the cognitive assessment because of visual, hearing, or upper‐limb impairments were excluded. Of these, 1079 completed the MMSE assessment. Participants with missing or nonevaluable CDT data (*n* = 5) and one participant with an implausible reported age were excluded, resulting in a final analytic sample of 1073 participants. Sampling flow is shown in Supplementary Figure 1.

### 2.2 Variables

#### 2.2.1 The CDT

The CDT was administered using a predrawn circle format. Participants were given a sheet of paper with a predrawn circle (9 cm in diameter) and were instructed as follows: (1) Inside the circle, please draw the hours of a clock as they normally appear, (2) place the hands of the clock to represent the time “11:10.” Drawings were evaluated using the 6‐point scoring system developed by Shulman et al. [13], where *lower* scores indicate better cognitive performance. All CDT drawings were independently scored by two trained raters with prior experience scoring MMSE assessments. While neither rater had previous experience with the CDT, the raters received a standardized training session based on a detailed scoring manual developed by the research team. The manual included illustrative examples representing each category of the Shulman scoring system. Example cases were reviewed collectively and discussed to ensure a common understanding of the scoring criteria before formal scoring commenced. Discrepancies between the two scores were resolved by an expert adjudicator (the first author), and the adjudicated score was used in subsequent analyses. The distribution of CDT score is illustrated in Supplementary Figure 2.

#### 2.2.2 The MMSE

The MMSE is a global measure of cognitive function, covering orientation, registration, attention and calculation, recall, and language abilities [14], and has been translated into several languages, including Vietnamese [15, 16, 17]. In this study, we used a Vietnamese version that had been used in a previous study conducted in Vietnam [15]. Scores range from 0 to 30, with higher values reflecting better cognitive performance. While the MMSE is widely used internationally, prior studies have demonstrated that its performance in Vietnam is strongly influenced by educational attainment. Specifically, Leggett et al. [15] and Bich et al. [16] found that the conventional cutoff (≤ 23) tends to overestimate cognitive impairment among individuals with no or limited formal education, and Miyawaki et al. [17] used a lower threshold (≤ 21) in a population with generally low educational attainment. Therefore, in this study, sensitivity analyses were conducted using alternative MMSE thresholds (≤ 21, ≤ 22) to examine whether the findings were sensitive to the choice of MMSE threshold.

#### 2.2.3 Sociodemographic Variables

Data on age (in years), sex (male or female), and educational attainment were obtained at baseline using a structured questionnaire. Educational attainment was assessed by asking participants to report the highest level of education completed: No formal schooling, less than primary school, primary school completed, secondary school completed, high school completed, college/university completed, or postgraduate degree. For analysis, these categories were grouped into four levels (less than primary education, completed primary school, secondary education, and higher education). In the Vietnamese educational system, primary education comprises 5 years, followed by 4 years of lower secondary education and 3 years of upper secondary education.

### 2.3 Statistical Analysis

#### 2.3.1 Inter‐Rater Reliability of the CDT

Inter‐rater reliability of the initial ratings was evaluated using the intraclass correlation coefficient (ICC) based on a two‐way mixed‐effects, absolute‐agreement model [ICC (3,1)], treating the two rating occasions (first and second rater) as fixed measurement conditions. In addition, the percentage of exact agreement was calculated to quantify categorical agreement between raters.

#### 2.3.2 Construct Validity of the CDT Relative to the MMSE

Construct validity of the CDT was examined by assessing its correlation with the MMSE. As CDT scores were not normally distributed, Spearman's rank correlation coefficient (*ρ*) was calculated to evaluate the monotonic association between CDT and MMSE scores. A significant negative correlation was expected, as higher CDT scores indicate poorer performance.

#### 2.3.3 Known‐Groups Validity of the CDT Across Educational Levels

Known‐groups validity was evaluated by comparing CDT scores across educational groups using the Kruskal–Wallis test. We hypothesized that participants with lower educational attainment would exhibit higher CDT scores, reflecting poorer cognitive performance.

#### 2.3.4 Concurrent Validity Relative to MMSE Performance

Concurrent validity was evaluated by examining the discriminatory performance of the CDT relative to MMSE performance using ROC analysis, sensitivity, specificity, predictive values, and by assessing agreement between CDT‐ and MMSE‐based classifications using Cohen's *κ*. MMSE scores ≤ 23, a commonly used cutoff in community‐based studies, were used as the reference classification. This threshold was used as an operational definition of low MMSE performance for comparison purposes rather than as a clinical diagnostic criterion. Sensitivity analyses were conducted using alternative MMSE thresholds of ≤ 21 and ≤ 22. Receiver operating characteristic (ROC) analysis was performed to calculate the area under the curve (AUC) and 95% confidence intervals (CIs). While the usual cutoff for CDT is ≥ 3 [13] the optimal CDT cutoff point among this population was investigated by maximizing the Youden index (sensitivity + specificity − 1). In addition, the level of agreement between CDT‐based and MMSE‐based classifications (impaired vs. nonimpaired) was evaluated using cross‐tabulation and Cohen's *κ* coefficient. The proportion of observed and expected agreement was also reported. We then tested alternative MMSE thresholds (≤ 21, ≤ 22) and calculated agreement indices (Cohen's *κ*) and ROC curves to examine whether the findings were sensitive to the choice of MMSE threshold. Sensitivity, specificity, and overall classification accuracy were reported for each cutoff condition.

To examine whether the discriminatory performance of the CDT varied by educational attainment, we additionally conducted education‐stratified ROC analyses. Participants were stratified into four educational groups (less than primary education, primary, secondary, or ≥ high school), and separate ROC curves and AUCs were calculated within each stratum. These analyses were performed to explore potential heterogeneity in the concurrent validity of the CDT across education levels and were considered exploratory. To facilitate visual interpretation of the relationship between CDT and MMSE performance, jittered scatter plots of MMSE scores across CDT score categories were generated and stratified by educational attainment (Supplementary Figure 3).

### 2.4 Ethics Approval

The KHCS protocol was approved by the institutional review boards of the National Center for Global Health and Medicine (currently the Japan Institute for Health Security) (approval number: JIHS‐S‐003172), the Pasteur Institute in Nha Trang (approval number: 02/2019/HDDD‐IPN), and the University of Tsukuba (approval number: 2108), with all participants providing written informed consent.

## 3 Results

### 3.1 Characteristics of the Study Participants

Table 1 summarizes the sample characteristics and test results. Mean age was 58.6 (standard deviation [SD] = 2.6), and male participants comprised 41.8% of the participants. Educational attainment varied: 8.7% had no or incomplete primary education, 30.1% had completed primary school, 33.5% had completed secondary school, and 27.8% had attained high school or higher education. The mean MMSE score was 26.7 (SD = 2.6), and 11.4% of the participants had low MMSE performance based on the cutoff of ≤ 23. The mean CDT score was 2.3 (SD = 1.1), with values ranging from 1 to 6. As shown in Supplementary Figure 2, the distribution was skewed toward lower scores: 26.7% of participants scored 1, 31.8% scored 2, 28.0% scored 3, and 10.3% scored 4. Fewer than 4% scored 5 or 6. Using the commonly applied cutoff in which scores of 1–2 indicate normal performance and scores of 3–6 indicate impairment, 41.6% were classified as impaired based on CDT scoring. The clinical characteristics of the study population are summarized in Supplementary Table 1. These data indicate that the present study was conducted in a community‐based population with a substantial burden of common chronic diseases.

**TABLE 1:** Characteristics of the study participants (*N* = 1073).

	*n* (%)/mean [SD]
Age (years)	58.6 [2.6]
Sex	
Male	449 (41.8%)
Female	624 (58.2%)
Education	
Less than primary education	93 (8.7%)
Primary	323 (30.1%)
Secondary	359 (33.5%)
≥High school	298 (27.8%)
CDT score	2.3 [1.1]
MMSE score	
Mean [SD]	26.7 [2.6]
MMSE ≤ 23	122 (11.4%)

### 3.2 Inter‐Rater Reliability

Inter‐rater reliability and construct validity are summarized in Table 2. Across the 1060 drawings with valid paired ratings (13 had a missing rating from one rater), the two independent raters showed an exact agreement rate of 64.6%. Treating the two rating occasions (first and second rater) as fixed measurement conditions, the ICC was 0.79 (95% CI: 0.76–0.81), reflecting good inter‐rater reliability. The average‐measures ICC was 0.88, indicating excellent reliability when rater scores are averaged or adjudicated. These results confirm that the CDT scoring protocol demonstrated adequate consistency across trained raters.

**TABLE 2:** Inter‐rater reliability and construct validity of the Clock Drawing Test.

Measure	Result	95% CI/*p*‐value	Interpretation
Inter‐rater reliability			
Exact agreement (%)	64.6%		Moderate agreement between raters
ICC (3,1)—single measures	0.79	0.76–0.81	Good inter‐rater reliability
ICC (3,k)—average measures	0.88	0.86–0.90	Excellent reliability when averaged/adjudicated
Construct validity			
Spearman's correlation (CDT vs. MMSE)	−0.30	*p* < 0.001	Significant negative correlation supporting construct validity

### 3.3 Construct Validity

There was a significant negative correlation between CDT and MMSE scores (Spearman's *ρ* = −0.30, *p* < 0.001), indicating that higher CDT scores (worse performance) were associated with lower MMSE scores. This finding provides some support for the construct validity of the CDT in relation to cognitive performance.

### 3.4 Known‐Groups Validity

Known‐groups validity and concurrent validity of CDT relative to MMSE performance are summarized in Table 3. The Kruskal–Wallis test indicated significant differences in CDT scores across education groups (*χ* ^2^(3) = 129.7, *p* < 0.001). Participants with lower educational attainment had higher CDT scores (worse performance). These results support the known‐groups validity of the CDT, reflecting expected differences in cognitive performance by education level.

**TABLE 3:** Known‐groups validity of the Clock Drawing Test and its concurrent validity relative to MMSE‐based classifications.

Measure	Result	95% CI/*p*‐value	Interpretation
Known‐groups validity			
Kruskal–Wallis test across education groups	*χ* ^2^(3) = 129.7	*p* < 0.001	CDT scores differ significantly by education level
Concurrent validity relative to MMSE performance (MMSE ≤ 23)			
AUC (CDT predicting MMSE impairment)	0.68	0.64–0.73	Modest discriminative ability
Sensitivity (cutoff ≥ 3)	67%		Balanced sensitivity
Specificity (cutoff ≥ 3)	62%		Moderate specificity
Overall accuracy	63%		Moderate classification performance
Positive predictive value (PPV)	18%		Low PPV; many false positives
Negative predictive value (NPV)	94%		High NPV; good for ruling out impairment
Cohen's *κ*	0.13	SE = 0.02, *p* < 0.001	Slight agreement with MMSE classification

Abbreviations: AUC: area under curve; CDT: Clock Drawing Test; CI: confidence interval; MMSE: Mini‐Mental State Examination.

### 3.5 Concurrent Validity Relative to MMSE Performance

ROC analysis revealed that the CDT had a modest ability to discriminate low MMSE performance defined by MMSE ≤ 23, with an AUC of 0.68 (95% CI: 0.64–0.73) (Figure 1). Using the cutoff of ≥ 3, the sensitivity and specificity were 67% and 62%, respectively, with an overall accuracy of 63%. The positive and negative predictive values were 18% and 94%, respectively. Agreement between CDT‐based and MMSE‐based classifications was 62.4%, yielding a Cohen's *κ* of 0.13 (SE = 0.02, *p* < 0.001). ROC analyses stratified by educational level showed heterogeneity in CDT performance (Figure 2). The AUC was lowest in participants with less than primary education (0.52), whereas modest discrimination was observed in the primary (0.65), secondary (0.67), and ≥ high school groups (0.63).

**FIGURE 1:**
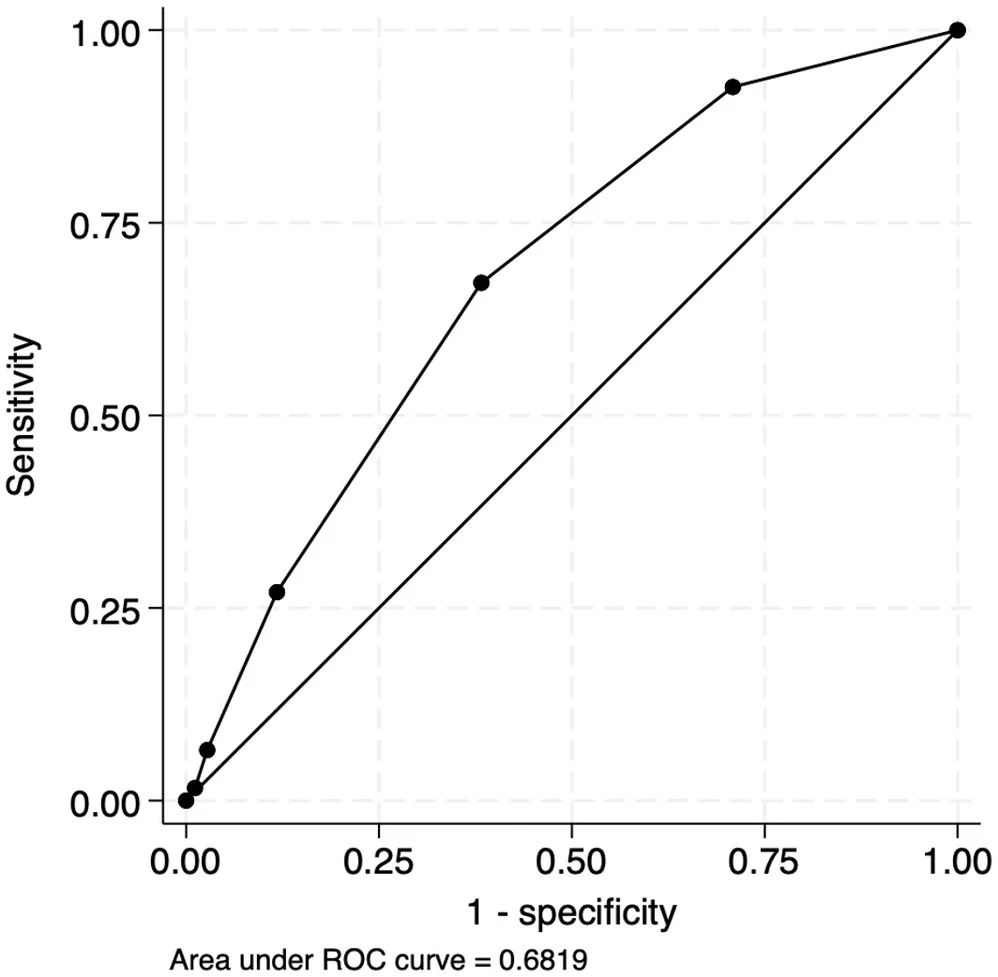
ROC curve for CDT in identifying low MMSE performance.

**FIGURE 2:**
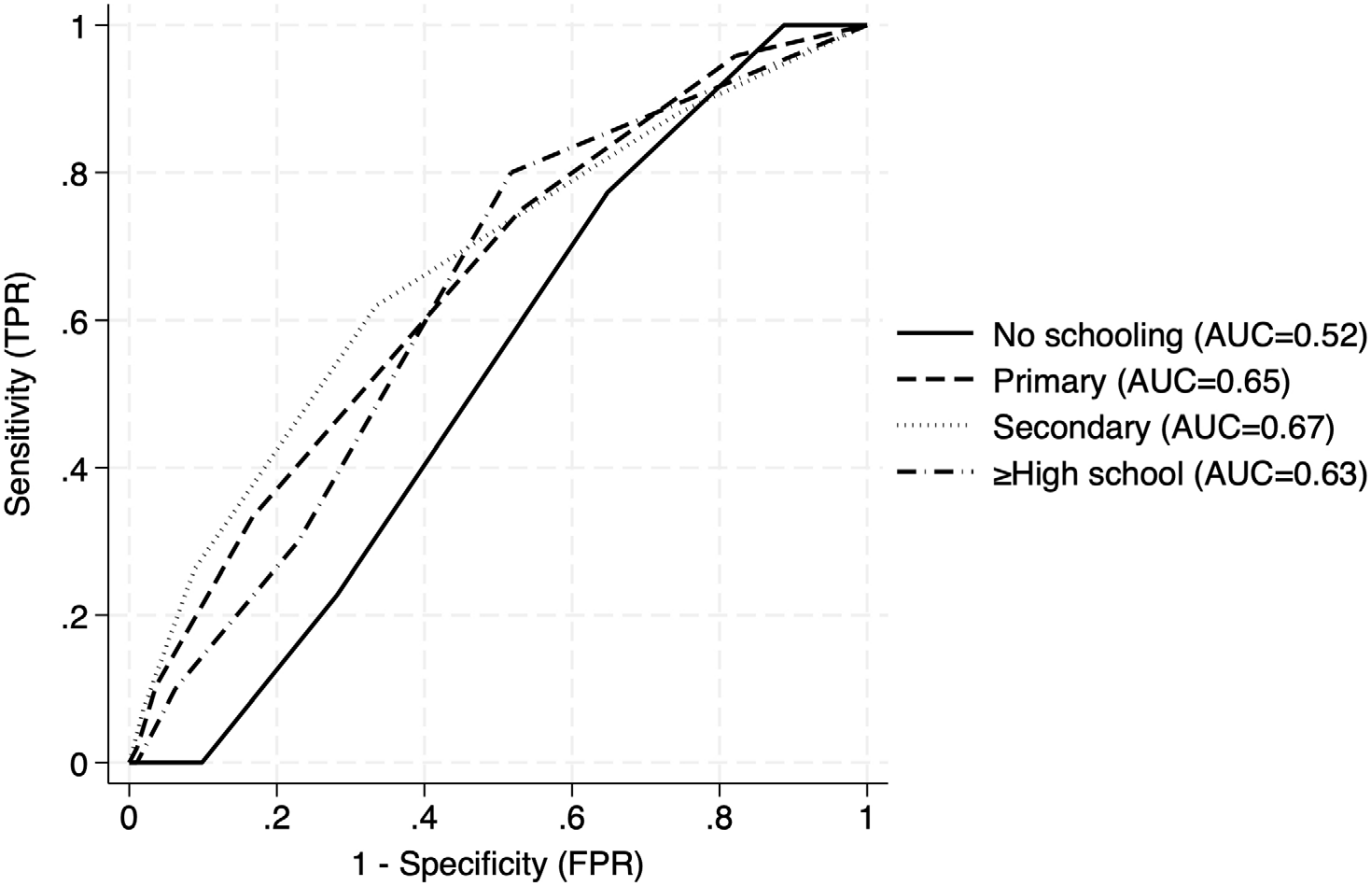
ROC curves for CDT in identifying low MMSE performance stratified by education.

Sensitivity analyses using alternative MMSE thresholds (≤ 21 and ≤ 22) did not improve agreement with CDT‐based impairment. When applying MMSE ≤ 21 as the criterion, sensitivity and specificity of CDT ≥ 3 were 74.5% and 60.1%, respectively, with low positive predictive value (8.5%) and high negative predictive value (97.9%), with slight agreement between classifications (*κ* = 0.07). Similarly, using MMSE ≤ 22 as the cutoff yielded sensitivity of 72.6% and specificity of 60.7%, with a positive predictive value of 12.0% and a negative predictive value of 96.8%, with slight agreement (*κ* = 0.10). These results indicate that concurrent validity analyses using alternative MMSE thresholds of ≤ 21 and ≤ 22 yielded patterns similar to those observed with the primary threshold of ≤ 23. Thus, the main findings were robust to the choice of MMSE cutoff used as the operational definition of low MMSE performance.

## 4 Discussion

This study evaluated the reliability and validity of the CDT among community‐dwelling older adults in rural Vietnam, focusing on its concurrent validity relative to MMSE performance in a community‐based setting. Overall, the CDT demonstrated acceptable inter‐rater reliability, while its concurrent validity relative to MMSE performance was modest and its performance varied by educational attainment.

Inter‐rater reliability was good, with a single‐measure ICC (3,1) of 0.79 and an average‐measures ICC of 0.88, indicating consistent application of the scoring protocol by trained raters. Construct validity was supported by a significant negative correlation between CDT and MMSE scores. The magnitude of this association (*ρ* = −0.30) was weaker than that reported in some earlier studies. For example, a study of 63 US dementia or Alzheimer's disease patients reported a stronger Pearson's correlation (*r* = −0.476) between CDT and MMSE scores [18]. In clinical samples with greater variation in cognitive performance and more severe impairment, stronger associations are expected, whereas our relatively young, community‐based sample likely had a narrower range of cognitive scores. Although the exact agreement rate between raters was 64.6%, the ICC indicated good overall reliability. Exact agreement is a stringent measure for ordinal rating scales because even disagreements between adjacent categories are counted as complete disagreement. The Shulman scoring system also requires qualitative judgment, particularly for borderline or atypical drawings that do not fit neatly into predefined categories. Differences in educational exposure, familiarity with formal testing, or experience with analog clocks may have contributed to such drawings in this population and made them more difficult to classify consistently.

Known‐groups validity analyses showed clear educational gradients, with poorer CDT performance among participants with lower educational attainment. This pattern is consistent with prior evidence suggesting that performance on visuospatial and executive tasks is influenced by educational exposure and familiarity with formal testing, and it underscores the importance of interpreting CDT in light of educational context [9, 19].

Concurrent validity analyses demonstrated modest discriminatory ability when using MMSE ≤ 23 to define cognitive impairment (AUC = 0.68). A cutoff of ≥ 3 yielded a reasonable balance between sensitivity (67%) and specificity (62%), with a high negative predictive value (94%) but a low positive predictive value (18%). This low positive predictive value is likely attributable, at least in part, to the low prevalence of MMSE‐defined cognitive impairment in this community‐based sample, as predictive values are known to be strongly influenced by outcome prevalence. Agreement between CDT‐based and MMSE‐based impairment classifications was only slight (*κ* = 0.13), suggesting that the two tests identify different subsets of individuals. This modest agreement is consistent with prior evidence that the CDT and MMSE assess overlapping but distinct cognitive domains: the MMSE focuses on orientation, memory, and language, whereas the CDT places greater demands on visuospatial organization and executive functioning [18, 20, 21]. Neuropsychological analyses have demonstrated that CDT errors are more sensitive to executive dysfunction than to memory decline, leading to weak correlations with MMSE in nonclinical samples [22]. Studies further show that some individuals exhibit impaired CDT performance despite normal MMSE scores, and vice versa [23, 24, 25]. Taken together, the slight agreement observed in our study may partly reflect differences in the cognitive domains assessed by the two instruments, although limitations in CDT performance in this population may also have contributed.

Education‐stratified ROC analyses revealed substantial heterogeneity in CDT performance. Discriminatory accuracy was lowest among participants with less than primary education (AUC = 0.52) and modest among participants with primary, secondary, or higher education (AUCs = 0.63–0.67). This pattern closely mirrors the findings of Lourenço et al. [9], who examined older Brazilian adults with low educational attainment using multiple CDT scoring systems, including the Shulman method. Although all scoring systems demonstrated excellent inter‐rater reliability (ICC = 0.94), their discriminative performance was modest (AUCs = 0.66–0.68) and was strongly influenced by educational attainment. Notably, older adults with minimal schooling frequently received abnormal CDT scores despite no clinically confirmed cognitive impairment. The authors concluded that education exerted a “significant and systematic impact” on CDT interpretation, and that changing scoring systems alone did not mitigate this effect. Consistent with the quantitative findings, Supplementary Figure 3 shows substantial overlap in MMSE scores across CDT score categories within each educational group. Although higher CDT scores generally corresponded to lower MMSE scores, considerable variability in MMSE performance remained within each CDT category. This overlap helps explain the modest correlation between the two measures and the modest concurrent validity of the CDT relative to MMSE performance. Taken together, these findings suggest that, in community‐based LMIC settings, CDT discrimination may be shaped by multiple factors, including educational exposure and familiarity with formal testing.

Although the CDT requires only a short administration time and minimal verbal instruction, its practical advantages may vary across populations. Successful completion of the task may be influenced by educational attainment, familiarity with analog clocks, drawing tasks, and broader cultural context. In addition, while administration is straightforward, consistent scoring requires appropriate training and standardized scoring procedures. In the present study, two trained raters independently scored each clock drawing, and discrepancies were resolved through adjudication to maximize scoring reliability for research purposes. This procedure was adopted to ensure methodological rigor and should not be interpreted as a necessary requirement for routine community‐based use. Nevertheless, the observed inter‐rater disagreement indicates that scoring feasibility should be considered when implementing the CDT in large‐scale epidemiological surveys.

The findings should be interpreted in light of certain limitations. First, concurrent validity was evaluated relative to MMSE performance rather than against a clinical diagnosis of cognitive impairment. MMSE scores were dichotomized at ≤ 23 as an operational definition of low MMSE performance, but MMSE results are influenced by educational attainment and cultural context. Accordingly, this threshold should not be interpreted as a clinical diagnostic category. Nevertheless, the overall pattern of concurrent validity remained consistent in sensitivity analyses using alternative MMSE thresholds of ≤ 21 and ≤ 22. Moreover, because the CDT and MMSE assess partially overlapping but distinct cognitive domains, the agreement between the two tests should not be interpreted as a definitive indicator of diagnostic accuracy. Second, although additional clinical characteristics of the study population are provided in Supplementary Table 1, physician‐confirmed diagnoses of dementia or mild cognitive impairment, functional status, Parkinsonism, and literacy were not assessed in this cohort. Therefore, we were unable to examine whether the performance of the CDT differed according to specific neurological or clinical conditions. Future studies should incorporate physician‐confirmed incident dementia to more accurately evaluate the predictive validity of the CDT. In addition, although the study protocol excluded individuals with visual, hearing, or upper‐limb impairments that precluded cognitive testing, these exclusions were recorded as field notes during data collection and were not retained as structured variables in the analytical dataset. Consequently, the number of participants excluded for these reasons could not be reported separately in the participant flow diagram. Third, the study population comprised late middle‐aged to young‐old adults rather than the substantially older populations typically targeted in dementia screening research. This relatively young age distribution may have contributed to the low prevalence of MMSE‐defined low cognitive performance, the restricted range of cognitive scores, and consequently the modest discriminatory performance and low positive predictive value observed for the CDT. Accordingly, the findings may not be directly generalizable to older populations, particularly adults aged 75 years or older, among whom dementia and clinically relevant cognitive impairment are more prevalent. Additionally, spectrum effects, where test performance decreases in samples with restricted disease severity, may have contributed to the modest AUC, as the sample consisted of relatively young adults with limited variation in cognitive severity. Fourth, we did not assess intra‐rater reliability; therefore, the stability of repeated scoring by the same evaluator could not be confirmed. Fifth, we did not evaluate test–retest reliability, which would provide information on the temporal stability of CDT scores.

## 5 Conclusions

The CDT demonstrated modest concurrent validity relative to MMSE performance in this rural Vietnamese population, with discriminatory performance varying by educational background. Although a high negative predictive value was observed, its implications for cognitive screening remain uncertain. Future studies using clinically confirmed outcomes are needed to establish the utility of the CDT for cognitive screening, refine context‐appropriate cutoffs, and improve the interpretation of CDT scores in LMIC epidemiological surveys.

## Author Contributions

A.Y., T.T.P.P., C.Q.N., and Y.I. contributed to the study conception. Methodological design was developed by A.Y. and Y.I., and A.Y. conducted the statistical analyses. The initial draft of the manuscript was prepared by A.Y., T.T.P.P., C.Q.N., and A.I. Critical revisions were provided by D.C.P., M.H., T.T.H., H.T.D., T.M., and Y.I. Funding was secured by H.T.D., T.M., and Y.I. All authors reviewed and approved the final manuscript.

## Funding

This work was supported by several funding sources, including internal research funds from the National Center for Global Health and Medicine (Y.I.: 19A06, 22A02) and the Japan Institute for Health Security (T.M.: 25A01), as well as the Pfizer Health Research Foundation (Y.I.) and Grants‐in‐Aid for Scientific Research (KAKENHI) from the Japan Society for the Promotion of Science (Y.I.: 19K20536, 23H03638; AY: 21J01171, 23K16371). A.Y. also received support through a Research Fellowship for Young Scientists from the Japan Society for the Promotion of Science. The funding bodies had no role in the design, analysis, or interpretation of the study, and the authors report no financial or personal relationships that could have influenced the work.

## Ethics Statement

This study adhered to the principles outlined in the Declaration of Helsinki. Ethical approval was obtained from the Research Ethics Committee of the Japan Institute for Health Security (formerly the National Center for Global Health and Medicine; approval no. JIHS‐S‐003172), as well as from the corresponding ethics committee at the Pasteur Institute Nha Trang (approval no. 02/2019/HDDD‐IPN) and the University of Tsukuba (approval no. 2108).

## Consent

Written informed consent was obtained from all participants prior to their inclusion in the study.

## Conflicts of Interest

The authors declare no conflicts of interest.

## Supporting information


**Supplementary Figure 1.** Flowchart of study participants.
**Supplementary Figure 2.** CDT score distribution.
**Supplementary Figure 3.** Distribution of MMSE scores according to CDT score, stratified by educational attainment.
**Supplementary Table 1.** Clinical characteristics of the participants (*N* = 1073).

## Data Availability

The dataset analyzed in this study cannot be made publicly accessible due to confidentiality considerations and the possibility of identifying individual participants. The data include sensitive information, such as personal health status, lifestyle characteristics, and early‐life exposures, and are linked to specific study sites, which may increase the risk of re‐identification. In line with decisions by the relevant ethics committees in Japan and Vietnam, public data sharing is therefore restricted. Nevertheless, the data may be provided upon reasonable request to the corresponding author.
