# Supplementary material for: Clock Drawing Test Performance in Rural Vietnam: Reliability and Concurrent Validity Relative to the Mini‐Mental State Examination in a Community‐Based Sample
Source: Geriatr Gerontol Int. 2026 Nov 3;26(11):e70876. doi: 10.1111/ggi.70876 (PMC13728975; doi:10.1111/ggi.70876)
Supplement: Supplementary file 1 [file GGI-26-0-s001.docx]

**Supplementary Figure 1.** Flowchart of study participants

**Supplementary Figure 2.** CDT score distribution

**Supplementary Figure 3.** Distribution of MMSE scores according to CDT score, stratified by educational attainment.

**Supplementary Table 1.** Clinical characteristics of the participants (N=1,073)

|  | n (%)/mean [SD] |
| --- | --- |
| Hypertension, n (%) | 619 (57.7) |
| Diabetes mellitus, n (%) | 131/1,069 (12.3) |
| History of cardiovascular disease or stroke, n (%) | 193 (18.0) |
| Depressive symptoms (CES-D ≥9), n (%) | 58 (5.4) |
| Body mass index, mean [SD], kg/m² | 23.2 [3.0] |

Hypertension was defined as a mean systolic blood pressure ≥140 mmHg, a mean diastolic blood pressure ≥90 mmHg, or current use of antihypertensive medication. Diabetes mellitus was defined as a fasting plasma glucose level ≥7.0 mmol/L or current use of antidiabetic medication or insulin. Diabetes status was unavailable for four participants because of missing glucose and medication data. History of cardiovascular disease or stroke was based on self-report of a previous diagnosis of heart disease (including heart attack or chest pain due to cardiovascular disease) or stroke. Depressive symptoms were defined as a score of ≥9 on the 11-item Center for Epidemiologic Studies Depression Scale (CES-D). Body mass index was calculated as weight (kg) divided by height squared (m²).
